# Editorial

**Published:** 1844-12-28

**Authors:** 


### THE MEDICAL EXAMINER.

### PHILADELPHIA, DEC. 28, 1844.

By an advertisement of the publishers on the cover of the present number of the MEDICAL EXAMINER, it will be seen that it is hereafter to appear in a greatly enlarged form. Seven years have now elapsed since its commencement, and in that comparatively long period for an American periodical, how many contemporaries have sprung into existence; and alas! how many, like Jonah's gourd, have withered and died! Humble as have been its pretentions, the MEDICAL EXAMINER, with two or three honorable exceptions, is now the oldest Medical Journal in the country. The substantial support which it has received during so long a time, and especially within the last year, in the opinion of the publishers, justifies greater out-lay, and increased efforts for the benefit of its patrons. In these views the editor concurs, and cheerfully undertakes to perform his part in the new scheme. The proposed enlargement will afford greater scope for his pen, and what is more important, allow of the insertion of many valuable original articles, not adapted to its present restricted limits; as well as permit more extended notices of new works, and the current medical literature of the day.

Without being invidious, it may be remarked that monthly publications are better adapted on several accounts, for the dissemination of solid information, than those of a more ephemeral character, whilst they may well subserve all the useful purposes of elaborate quarterlies. From the number of the monthly Medical Journals in the United States, compared with those of either longer or shorter periods, this would seem to be fully sanctioned by experience. Whether another may be profitably added to the list, time must determine: for ourselves we entertain no doubts on the subject. Our subscribers, we hope, will see in the increased size and value of the Journal, without any increase of the price, additional ground for their confidence and support.

According to the plan proposed by the publishers, the Examiner will be one of the *cheapest* Medical periodicals extant, and the editor hopes, from the abundant means at his command, to make it one of *the best.* Already he is promised contributions by some of the ablest and most experienced writers in the profession; and he trusts the number will be greatly augmented—for that purpose, he solicits the co-operation of his brethren throughout all parts of this widely extended country. Interesting cases; accounts of epidemic and endemic diseases; able discussions of moot points; temperate remarks on errors in doctrines and practice; notices of new remedies, and of the properties of indigenous medicines; in short, well written communications on all subjects likely to interest the profession and enlarge the boundaries of the science, will be gratefully received, and in every instance, the writers will be furnished with a copy of the number in which their contributions are inserted.

---

### LONDON LANCET.

Some weeks since, we called the attention of the profession to the mutilated form in which this Journal was re-published in this country. Since then, we learn that it has been altogether discontinued by those who were engaged in the enterprise. We are not surprised at this. They doubtless found it an unprofitable job. The truth is, or we are greatly mistaken, there are few foreign journals of any kind, and especially such as pertain to medicine, that will bear re-publication in this country.

Of the British Medical Journals, the larger, which are exclusively devoted to medical science, are so heavy and costly, that one only (the Medico-Chirurgical) has obtained a subscription list adequate to the expense of re-publication; while the smaller are so occupied with personal disputes and matters of local interest, as to greatly lesson their attractions for distant readers.

London has three weekly medical journals, conducted with great ability, viz.: "*The London Medical Gazette*," "the *London Lancet*," and the "*London Times.*" Of these, the two latter occupy antagonist positions, and may be said to represent opposite parties or factions of the profession in that great metropolis. Accordingly we find, mixed up with much valuable matter, a large amount of a different stamp—very interesting, no doubt, to the physicians and surgeons of London, but about as much so to us in the United States, as our Corporation, County and State politics, are to our brethren on the other side of the Atlantic. The discussions on "Sir James Graham's Bill," "Medical Reform," the fitness or unfitness of the last appointment at St. Bartholomews', St. Thomas', at Guy's, "Movement of the Profession," "Great meeting at Leeds," "at Manchester," &c. &c., with many other things proper to London, are all very well in their place, but to American physicians they are nearly as cabalistic as "the secret science of the Jews."

---

The following sensible remarks are extracted from one of our cotemporaries. We rejoice to see in it the evidence of a desire to cultivate good feeling among those who ought never to indulge any other. If our brethren elsewhere will look to the example afforded by Philadelphia physicians in this respect, they will discover how much individual happiness and prosperity is promoted by a regard for professional honor and respect.

### PROFESSIONAL COURTESY.

Courtesy, which is but an expression of kindness, is the principle ingredient that sweetens the toils and cares and conflicting interests with which life abounds. How cheerless would be our prospects, what the number of impediments to obstruct our path, how unhappy our journey of life, were we to give expression only to those tumultuous feelings which arise from concentrating every thought upon self! Courtesy is the beauteous ornament of social life, and as such has always been cultivated by the refined and educated, and regarded as an indispensable requisite for the perfection of social intercourse. The exercise of courtesy is equally demanded from all, whether it be among those in the kindly intercourse of friendship, or such as are found in the less refined but more necessary meetings of business. Indeed it is more needed under circumstances where self-interest is liable to smother the kind feelings of our common nature, and still more is it required among those whose similarity of pursuits bring their talents and professional abilities in contact and competition.

If there is one class where this especially is the case, and where at the same time courtesy might with reason be looked for, and in whose character it would almost seem to be an indispensable ingredient, it is among the members of the medical profession.

Kindness is the habitual exercise of the mind with those whose necessary occupation is to afford relief to others; and as medical men are of all people those whose opportunities for such exercise are the most frequent, it is among them that we should expect to see it in its most ample manifestation—extending its influence beyond the immediate sphere of their professional duties, and embracing all who are honorably united in their efforts to mitigate the physical evils of life. Kindness of feeling is the germ of real courtesy; an essential part of the latter must be true kindness. Courtesy, in its full signification, is not an artificial, hollow, unmeaning expression of consideration, it is in fact one expression of humility as opposed to self-importance or selfishness and pride. It has been often assumed, and then cannot be exhibited without borrowing the entire plumage. Thus the immortal bard:

> And then I stole all courtesy from heaven,
> And dress'd myself in such humility
> That I did pluck allegiance from all hearts.

A courteous demeanor towards each other might therefore be very naturally expected to be exercised among the professors of an art which is kindness itself in the most efficient manifestation.

Courtesy is indeed indispensable to the character of the physician, in whatever light it may be viewed. By courtesy among each other, their powers of doing good would be increased,—their mutual good will would lead to a frequent interchange of views on scientific subjects. If it is desired to place the profession of medicine in its most elevated rank, and elicit for it the esteem and admiration which are essentially its due, what more efficient method than to adorn it with that evidence of mutual respect among its members, which would infallibly mark them as superior to all petty rivalries and contemptible jealousies? If their individual happiness is to be promoted, what more powerful means than the constant exercise of mutual courtesy, which is but the external expression of benevolence? Point out the man that spurns all courtesy from his demeanor, and we will mark one where envy and hatred—fell destroyers of all happiness—are secretly gnawing, with the deadly poison of a viper's fang. Such an individual is unfit for the profession of the healing art.

It has been strangely asserted and most extensively believed, that medical men are a jealous set, destitute of courtesy among themselves, that they have no kind feelings towards each other; thus, somehow or other, charging these faults upon them as a class, and consequently making them the natural and necessary effects of the profession they have adopted. Now we believe no such thing. This jealousy is an excrescence—a fungous growth—and is no more to be charged upon the profession itself, than is arrant quackery. It is a part of the wickedness of our nature, and must be participated, more or less, by all men of whatever profession, precisely as charlatanism and imposture belong to men as a race, and not to the professors of the medical art—physic being only used as the medium of deception from its admitting of so great an amount of mystery in its application.

We have seen the strongest friendship exist between medical men, having its origin in professional intercourse; and it is a remarkable fact in the character of all, that an interminable conversation of the most agreeable nature imaginable will ensue upon their meeting either casually or upon professional business. Two doctors meeting and exchanging civilities are almost inseparable.

Finding, then, that the seeds of courtesy exist within the bosom of our fraternity, it needs nothing more than a proper cultivation to make them germinate and yield rich clusters of fair flowers, whose fragrance will spread far and wide—perhaps even unheeded and unknown in their remoteness, save in the mild and genial influence they produce.—*N. Y. Journal of Medicine.*

